# ASO Author Reflections: Gastric Venous Congestion After Total Pancreatectomy is an Underestimated Complication

**DOI:** 10.1245/s10434-023-13979-2

**Published:** 2023-08-10

**Authors:** Thomas F. Stoop, André von Gohren, Jennie Engstrand, Ernesto Sparrelid, Stefan Gilg, Marco Del Chiaro, Poya Ghorbani

**Affiliations:** 1 Division of Surgery, Department of Clinical Science, Intervention and Technology (CLINTEC), Karolinska Institutet at Karolinska University Hospital, Stockholm, Sweden; 2 Amsterdam UMC, Location University of Amsterdam, Department of Surgery, Amsterdam, The Netherlands; 3 Cancer Center Amsterdam, Amsterdam, The Netherlands; 4 Division of Surgical Oncology, Department of Surgery, University of Colorado Anschutz Medical Campus, Aurora, CO USA

## Past

A renewed interest in recent years has evolved for total pancreatectomy (TP) for various oncological, technical, and safety indications^1^ owing to improved surgical outcome and manageable metabolic insufficiencies with acceptably reduced quality of life.^2^ However, a rarely studied/mentioned complication after TP is gastric venous congestion (GVC), despite its seemingly high incidence of up to 28% and possible association with mortality.^3^

## Present

The present retrospective single-center study investigated the incidence, risk factors, management, and outcome of GVC after elective TP.^4^ The incidence of GVC was 21% among 268 consecutive patients who underwent TP. In most patients with GVC, the diagnosis was made during index surgery (93%) and managed with a (sub)total gastrectomy in 55% of patients. The clinical relevance of GVC was illustrated by the fact that intraoperative GVC was an independent predictor for major morbidity. Predictors for GVC were portomesenteric venous resection and left coronary vein ligation. Therefore, perioperative vigilance is required in these patients, lowering the threshold for surgical interventions [e.g., (sub)total gastrectomy or reconstruction of the gastric venous outflow] and eventually performing additional intra- and postoperative diagnostics (e.g., gastroscopy and indocyanine green fluorescence).

## Future

Considering the high incidence of GVC after TP and its clinical impact, prospective studies are needed on prevention, detection, and optimal management of GVC.^5^ Furthermore, there is need for standardization of terminology, including a severity-based classification for GVC to support clinical decision-making, prognostication, and research on management of GVC.
